# Association Between Atrial Fibrillation and Dementia: A Meta-Analysis

**DOI:** 10.3389/fnagi.2019.00305

**Published:** 2019-11-08

**Authors:** Md. Mohaimenul Islam, Tahmina Nasrin Poly, Bruno Andreas Walther, Hsuan-Chia Yang, Chieh Chen Wu, Ming-Chin Lin, Shuo-Chen Chien, Yu-Chuan Li

**Affiliations:** ^1^Graduate Institute of Biomedical Informatics, College of Medical Science and Technology, Taipei Medical University, Taipei, Taiwan; ^2^International Center for Health Information Technology (ICHIT), Taipei Medical University, Taipei, Taiwan; ^3^Research Center of Big Data and Meta-Analysis, Wan Fang Hospital, Taipei Medical University, Taipei, Taiwan; ^4^Department of Biological Sciences, National Sun Yat-sen University, Kaohsiung City, Taiwan; ^5^Department of Neurosurgery, Shuang Ho Hospital, Taipei Medical University, New Taipei City, Taiwan; ^6^TMU Research Center of Cancer Translational Medicine, Taipei, Taiwan; ^7^Department of Dermatology, Wan Fang Hospital, Taipei, Taiwan

**Keywords:** atrial fibrillation, dementia, cardiac disease, cardiac arrhythmia, stroke, hypertension

## Abstract

**Background:** A potential evidence from previous epidemiological studies remains conflicting findings regarding the association between atrial fibrillation (AF) and dementia risk. We, therefore, carried out a meta-analysis of relevant studies to investigate the magnitude of the association between AF and dementia risk.

**Methods:** We performed a systematic literature search of PubMed, EMBASE, and Google Scholar for potential studies between January 1, 1990, and December 31, 2018, with no restriction on the publication language. All potential studies were independently assessed by two reviewers. We only included observational studies that calculated the odds ratio (OR)/hazards ratio (HR) for dementia associated with atrial fibrillation. We first assessed the heterogeneity among study-specific HRs using the Q statistic and *I*^2^ statistic. We then used the random-effects model to obtain the overall HR and its 95% CI for all studies. We also tested and corrected for publication bias by funnel plot–based methods. The quality of each study was assessed with the Newcastle Ottawa Scale.

**Results:** A total of 16 studies with 2,415,356 individuals, and approximately 200,653 cases of incidence dementia were included in this study. Patients with AF had a greater risk of incidence dementia than those without AF (random-effect hazard ratio HR: 1.36, 95% CI: 1.23–1.51, *p* < 0.0001; *I*^2^ = 83.58). Funnel plot and Egger test did not reveal significant publication bias. However, limitations of the study included high heterogeneity and varying degrees of confounder adjustment across individual studies.

**Conclusion:** This study serves as added evidence supporting the hypothesis that AF is associated with an increased risk of dementia. More studies are needed to establish whether optimal treatment of AF can reduce or mitigate the risk of dementia.

## Introduction

Atrial fibrillation (AF) is one of the most common cardiac arrhythmia (e.g., affecting approximately 2.7–6.1 million people in the United States of America) (January et al., 2014; Mou et al., 2018), and is characterized by irregular RR intervals and the absence of distinct P waves (European Heart Rhythm Association et al., 2010). The prevalence of AF is expected to rise further in the near future (Heeringa, 2010). AF has been traditionally considered to be a potential risk factor of ischemic stroke, thromboembolism, heart failure, myocardial infarction, and death (You et al., 2012; Piccini et al., 2013). Although a handful of epidemiological studies reported that the risk of vascular dementia always increases after an ischemic insult, there are still knowledge gaps about AF related cognitive decline and dementia beyond aging and stroke (Gorelick et al., 2011; Ding and Qiu, 2018). In a meta-analysis, Kalantarian et al. (2013) reported that AF is associated with a higher risk of cognitive impairment and dementia (~40%), with or without a history of clinical stroke. Similarly, Santangeli et al. (2012) mentioned that patients with AF had a 1.42-fold increased risk of dementia compared to patients without AF.

Dementia is characterized by cognitive and behavioral deficits and several potential factors such as increasing age, apolipoprotein E gene isoform 4 (APOEε4), diabetes, dietary factors, traumatic brain injury, and multiple environmental factors are associated with incidence and progression of dementia (Polidori et al., 2012; Banik et al., 2015; Islam et al., 2016). However, it is now well-established that the link between AF and dementia is more complicated than previously suspected. These two diseases shared common modifiable risk factors, such as lifestyle factors (e.g., physical activity, smoking, and alcohol consumption), cardio-metabolic risk factors (e.g., overweight, hypertension, diabetics, high cholesterol) and pathophysiological pathways (e.g., systematic inflammation, cerebral small vessel diseases, cerebral hypo-perfusion) (Qiu and Fratiglioni, 2015; Dietzel et al., 2017). In addition, evidence from *in-vivo* studies suggests that long-term AF diminishes cardiac output and may precede and/or promote chronic cerebral hypo-perfusion and hypoxia. Therefore, it impairs the clearance and enhance the accumulation of amyloid-β peptides collection in cerebral vessels, and thus ultimately increases the risk of Alzheimer's disease (AD) (Bell and Zlokovic, 2009; Ihara and Washida, 2018). Moreover, previous studies showed that inflammation markers like C-reactive protein, interleukin (IL)-2, IL-6, IL-8, and monocyte chemoattractant protein (MCP)-1 are always incorporated with hyper-coagulation and endothelial dysfunction that finally lead to AF-related thromboembolism formation (Friedrichs et al., 2011). However, a higher level of systemic inflammations is responsible for blood-brain barrier (BBB) damage and cerebral microstructural changes; thus, it ultimately contributes to cognitive decline and dementia in patients with AF (Takeda et al., 2014).

We herein report the results of a systematic review and meta-analysis of a higher number of epidemiological studies that explored the relationship between AF and dementia risk. Our aim was to gauge precisely the nature and magnitude of the association between AF and dementia risk. We also investigated dementia risk based on region, study design and duration of AF (≥5 years and <5 years). However, correctly evaluate the extent of the association between AF and dementia risk may have immense clinical inferences for the identification, prevention, and treatment of dementia.

## Research Design and Methods

### Registration of Review Protocol

The protocol for this systematic review was registered in advance with PROSPERO (International Prospective Register of Systematic Reviews, no. **CRD42018117343**).

### Research Design

All the studies were included and excluded according to the Preferred Reporting Items for Systematic Reviews and Meta-Analyses (PRISMA) flow diagram recommended by the Cochrane library (Liberati et al., 2009). Since, only observational studies were considered for inclusion; we therefore considered the Meta-Analysis of Observational Studies in Epidemiology (MOOSE) guidelines for the meta-analysis of observational studies (Stroup et al., 2000; Poly et al., 2017; Wang et al., 2018) (Supplementary Table 1).

### Literature Search

Initially, we systematically search in PubMed, Scopus, and Web of Science for relevant studies published between 1 January 1990 and 1 October 2018 using the free text term “atrial fibrillation” AND “dementia risk” OR “vascular dementia” OR “Alzheimer risk” (Supplementary Table 4). In addition, to ensure comprehensiveness, reference lists of retrieved studies and previous review articles were searched for additional relevant studies. Finally, widely used referencing software, EndNote X7 (Thomson Reuters) was applied to check duplication.

### Study Selection Criteria

Two authors (MMI, TNP) who are expert in meta-analysis subsequently screened all the titles and abstracts for the studies included primarily in our meta-analysis. All the selected studies from this initial screening were then being considered for full-text review. Any disagreement during the screening stage was clarified by discussion with the other experts (HCY and CCW).

Studies were considered for inclusion in the meta-analysis if they fulfilled the following criteria:

(i). Types of studies
  Observational design study such as cohort, case-control, or randomized control trial (RCT).
(ii). Types of participants
  - An adult (aged ≥ 18 years) with atrial fibrillation (AF).
  - Included studies that reported AF and dementia risk and AF inclusion was confirmed through ECG and physician's diagnosis (ICD-9/10 code), and other standard codes at baseline and each follow-up. Furthermore, for all individuals, it also needed to be confirmed that the presence and onset date of AF was prior to the baseline period and continued during the follow-up periods. A study considered all participants with AF at baseline to have prevalent AF, and also those who developed AF during the follow-up before the diagnosis of any kind dementia.
  - Included studies that reported AF and dementia risk and screened cognitive function using the Mini-Mental State Examination (MMSE) and also provided information regarding the dementia diagnosis based on the DSM-III revised or DSM-IV criteria validated 3-step procedure. We also included studies that examined vascular dementia and AD according to the National Institute of Neurological Disorders and Stroke and Association Internationale pour la Recherch'e et l'Enseignement en Neurosciences (NINDS-AIREN) criteria, and National Institute of Neurological and Communicative Disorders and Stroke and the Alzheimer's Disease and Related Disorders Association (NINCDS-ADRDA) criteria, respectively.
  - Included studies that reported AF and dementia risk and dementia patients was identified by the International Classification of Diseases, Clinical Modification (ICD-9/10-CM) were also included in our study.
(iii). Type of intervention
  AF patient for at least 1 year or longer. Any study with at least a 1 year follow-up period was considered for inclusion. A valid control group was healthy patients without AF and dementia.
(iv). Type of outcome
  Primary outcome: Development of any kind of dementia in patients with AF.
  Secondary outcome: Development of AD in patients with AF.
  We excluded study that did not meet the following criteria: (1) study that was published as a case-report, editorial, review, or clinical trial; and (2) study that did not report dementia risk as their outcome.

### Data Extraction

Data abstraction was conducted by MMI and TNP who used a predefined, standardized guidelines, and data collection procedures. They used the Review Manager software (RevMan-5) to check data accuracy. The following information was garnered from the included studies: (a) method: study design, data collection period, study duration, number of study centers and location, study setting, study protocol, inclusion and exclusion criteria; (b) participants: total number of participants, total number of dementia patients, mean age of participants, age range, gender, percentage of gender, diagnostic criteria; (c) interventions: intervention, comparison, concomitant medications, comorbidities; (d) outcome: primary and secondary findings. Afterward, MMI and TNP garnered the effect size data (HR with 95% CI) from all included observational studies. We resolved disagreements by discussing with the main investigator (YCL).

### Assessment of Bias Risk

MMI and TNP utilized the Newcastle-Ottawa Scale (NOS) for evaluating the quality of each non-randomized study (cohort study). The NOS scale was used to address the participants selection, study comparability, and outcome or exposure assessment, with scores ranging from 6 to 9 (score 9 considered as no bias) (Stang, 2010) (Supplementary Table 2). Studies were then also assessed to be of good, fair, or poor quality (Donnelly et al., 2017). In addition, the methodological quality of the RCT was independently evaluated using the Cochrane collaboration's tool for measuring the potential risk of bias. The guidelines were also followed to determine whether trials took appropriate steps to minimize the risk of bias across six areas: (a) sequence generation, (b) allocation concealment, (c) blinding (study participants, personnel, and outcome), (d) incomplete outcome data, (e) selective outcome reporting, (f) other sources of bias. Finally, the risk of each study was classified into low, high, or unclear risk of bias (Higgins and Green, 2011). Moreover, the heterogeneity among study was determined by and statistic. Publication bias was also tested and corrected by the funnel plot-based method: Egger's regression test.

### Subgroup Analyses

A comprehensive subgroup analyses was conducted with type of study design (cohort or RCT), continents (Europe or North America), and methodological quality (good or fair).

### Statistical Analyses

The comprehensive meta-analysis software (CMA), version 3, was utilized to conduct statistical analyses. The overall hazard ratios (HRs) from individual studies were pooled using a random-effects model. Furthermore, forest plots were drawn to visually evaluate the results of pooling. AHR value > 1 indicates an increased risk of dementia, HR value 1 indicates no observed association, and HR value < 1 indicates a decreased risk of dementia. In case of statistical significance, *p* < 0.05. Furthermore, the heterogeneity in the results among the studies was calculated using the Higgins *I*^2^ which measures the percentage of the total variation across the included studies (Higgins and Thompson, 2002). The *I*^2^ was calculated as follows:

$$\begin{array}{l} I^{2}=\frac{\left(Q-df\right)}{Q}\times 100\% \end{array}$$

Here, Q is Cochran's heterogeneity statistic and df is the degree of freedom.

$$\begin{array}{l} \text{The formula }Q=\sum \left(w\times ES^{2}\right)-\frac{{\left[\sum \left(W\times \;ES\right)\right]}^{2}}{\sum W} \end{array}$$

where w is the individual study weights and w^*^ES is the weighted effect size that is calculated by multiplying each effect size by the study weight. Negative values of *I*^2^ are considered equal to zero. The values of *I*^2^ therefore lies between 0 and 100%. A value of 0% indicates no observed heterogeneity. However, a value of *I*^2^ at 25–50%, 50–75%, and more than 75% is considered as mild, moderate, and severe heterogeneity (Higgins et al., 2003). Moreover, tau-squared (τ^2^) was also calculated to see the extent of variation among the effects observed in different studies (between-study variance). It represents the absolute value of the true variance (heterogeneity) (Deeks et al., 2008). The equation of calculating τ^2^ is given by:

$$\begin{array}{l} \tau^{2}=\frac{Q-df}{\sum w_{i}-\frac{\sum w_{i}^{2}}{\sum w_{i}}} \end{array}$$

A visual exploration of the funnel plot was shown to evaluate publication bias. A two-sided *P* < 0.05 was considered statistically significant.

## Results

### Study Screening

**Figure 1:**
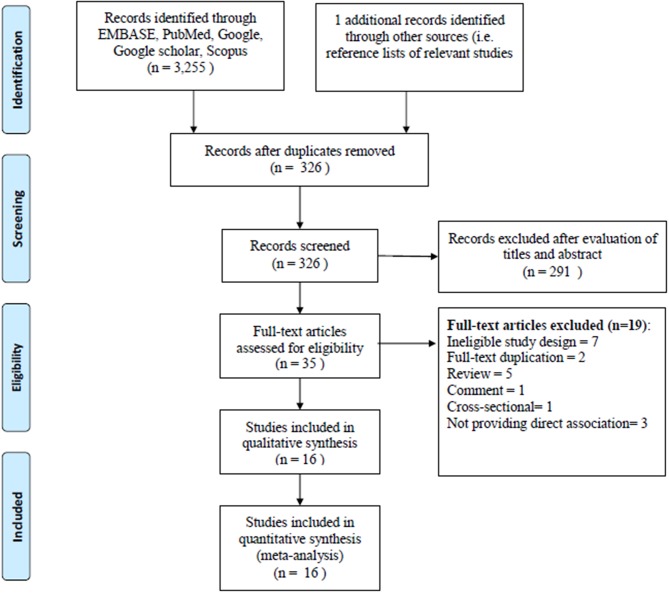
PRISMA flow diagram for study selection.

The literatures search of the electronic database yielded 3,256 articles. A total of 3,221 articles were excluded when we reviewed all the titles and abstracts; it is due to lack of adherence to our inclusion criteria. Thirty five articles were selected for the full-text revision and checked their reference lists for relevant articles, retrieving two additional articles. However, another 19 articles were excluded for non-adherence with the inclusion criteria. Furthermore, two articles were excluded for data overlap with another study. We also excluded three articles for not providing the associated risk of dementia with AF. Finally, 16 studies were included in our meta-analysis (Tilvis et al., 2004; Elias et al., 2006; Forti et al., 2006; Rastas et al., 2007; Peters et al., 2009; Bunch et al., 2010; Dublin et al., 2011; Marengoni et al., 2011; Marzona et al., 2012, 2016; Rusanen et al., 2014; de Bruijn et al., 2015; Liao et al., 2015; Singh-Manoux et al., 2017; Chen et al., 2018; Ding et al., 2018). The flow diagram of inclusion and exclusion information is presented in Figure 1.

### Study Characteristics

The study characteristics of the included 16 studies are presented in Table 1. Overall, 16 observational studies, comprising 2,415,356 individuals (200,653 dementia patients) were included in the meta-analysis. All the studies were published between 2004 (Tilvis et al., 2004) and 2018 (Chen et al., 2018). In this meta-analysis, 14 studies had a cohort study design (Tilvis et al., 2004; Elias et al., 2006; Forti et al., 2006; Rastas et al., 2007; Bunch et al., 2010; Dublin et al., 2011; Marengoni et al., 2011; Rusanen et al., 2014; de Bruijn et al., 2015; Liao et al., 2015; Marzona et al., 2016; Singh-Manoux et al., 2017; Chen et al., 2018; Ding et al., 2018) and two studies had an RCT design (Peters et al., 2009; Marzona et al., 2012). The mean age of patients for men and women was almost same; 20.43 to 100% of the sample population were men. The studies were conducted in diverse study populations with various comorbidities, and 10 studies included participants with type 2 diabetes; and hypertension (Supplementary Table 3). The minimum follow-up period ranged from 1.8 to 26.6 years. Moreover, most studies used standardized methods for identifying AF and dementia patients and had a low risk of bias. Most of the included studies were conducted in the Europe (Finland, Italy, Sweden, UK and Netherlands), four studies were carried out in the USA, and one in Asia (Taiwan). All the studies included AF and dementia patients based on a standard protocol.

**Table 1:** Characteristics of included studies.

**References**	**Country**	**Study design**	**% of male**	**No. of pts**.	**No. of dementia**	**Inclusion criteria**	**Exclusion criteria**	**AF diagnosis**	**Dementia diagnosis**	**Follow-up period (years, months)**	**Outcome measure**
Tilvis et al. (2004)	Finland	Cohort	26.39	629	629	Random sample of pts born in 1904,1909, and 1914	No specified	H&P, medical record	MMSE and CDR	5	HR = 2.88 (1.26–6.06)
Elias et al. (2006)	USA	Cohort	100	1,011	952	Male offspring of the Framingham heart study	Pts with dementia, prior stroke, females, decline examination	ECG, ECG-H, H&P	Neuropsychological tests approved by a panel of neurologists and neuropsychiatrists	30	HR = 4.01 (1.84–8.74)
Forti et al. (2006)	Italy	Cohort	37	431	36	Pts > 60 yrs. seeking medical advice for cognitive complaints	Pts with psychiatric disorder, Parkinson disease, epilepsy substance abuse	H&P	MMSE and neuropsychological tests	4	HR = 1.10 (0.40–3.03)
Rastas et al. (2007)	Finland	Cohort	20.43	1,106	339	Pts ≥ 85 y between 1991 and 1999	Pts who died before examination	ECG, ECG-H, medical records	DSM-III, MMSE	3.5	HR = 0.86 (0.50–1.47)
Marengoni et al. (2011)	Sweden	Cohort	NR	685	170	Pts > 75 years	Pts with dementia, prior stroke	H&P, medical records, ICD codes	DSM-III Revised	6	HR = 0.9 (0.50–1.70)
Peters et al. (2009)	UK	RCT	34.59	3,336	263	Pts > 80 yrs. with hypertension	Pts with dementia	Not specified	DSM-IV	1.8	HR = 1.03 (0.62–1.72)
Bunch et al. (2010)	USA	Cohort	60.1	37,025	1,535	Pts included in the Intermountain Healthcare System database	Pts with dementia	ICD codes	ICD codes	5	HR = 1.36 (1.13–1.63)
Dublin et al. (2011)	USA	Cohort	40	3045	572	Pts ≥ 65 yrs.	Pts with dementia, prior stroke	ICD codes	DSM-IV	6.8	HR = 1.38 (1.10–1.73)
Marzona et al. (2012)	Italy	RCT	70.3	31,506	890	Pts ≥ 55 yrs., history of cardiovascular disease or diabetes with end-organ damage	Pts with heart failure, substantial valvular disease, or uncontrolled hypertension	ECG, medical records	MMSE	4.7	HR = 1.30 (1.14–1.49)
Rusanen et al. (2014)	Finland	Cohort	37.6	1,510	127	Pts of the Cardiovascular Risk Factors, Aging and Dementia (CAIDE) study, Pts aged 65 to 79 yrs	Not properly examined, not properly followed-up	ICD codes, MRI/CT	ICD codes, DSM-IV, MMSE	7.8	HR = 2.61 (1.05–6.47)
Liao et al. (2015)	Taiwan	Cohort	55.9	665,330	56,901	AF with age ≥ 20 years and no history of dementia for at least 1 year preceding study enrollment	Not specified	ICD codes	ICD codes	15	HR = 1.42 (1.39–1.44)
de Bruijn et al. (2015)	Netherlands	Cohort	40.6	6,194	994	Pts with proper identification of AF, proper follow-up	Not properly screened for dementia, had previously dementia, lack of follow-up, missing data on AF	EEG and MEANS	MMSE, GMSS, and DSM-III revised	20	HR = 1.33 (0.99–1.78)
Marzona et al. (2016)	Italy	Cohort	47.48	1,627,631	134,837	≥65 yrs. pts with AF	Pts admitted to hospital for AF in the previous two years (2000–2001). Patients with incident dementia in the 2 years before entering the study	ICD codes	ICD codes	10	HR = 1.17 (1.12–1.23)
Singh-Manoux et al. (2017)	UK	Cohort	84.3	10,308	912	Pts with proper identification of AF and dementia, follow-up examination approximately every 5 years	No specified	ICD code and ECG	ICD code	26.6	HR = 1.87 (1.37–2.55)
Ding et al. (2018)	Sweden	Cohort	37.1	2,685	399	Pts aged >60 yrs.	Dementia at baseline and refused follow-up	ICD code	MMSE and DSM-III revised, NINDS-AIREN, NINCDS-ADRDA	9	HR = 1.88 (1.09–3.23)
Chen et al. (2018)	USA	Cohort	44	22,924	1,157	Pts aged >45 yrs., proper cognitive data	Dementia at baseline and refused follow-up	EEG and ICD code	DWRT, DSST, WFT, ARIC-NCS, MMSE, ICD code	20	HR = 1.23 (1.04–1.45)

*MEANS, Modular ECG Analysis System; ECG, Electrocardiography; MMSE, Mini-Mental State Examination; GMSS, Geriatric Mental State Schedule; DSM-III, Diagnostic and Statistical Manual of Mental Disorders (Third Edition Revised); ICD, International Classification of Diseases; CDR, Clinical Dementia Rating; NINDS-AIREN, National Institute of Neurological Disorders and Stroke and Association International pour la Recherch'e et l'Enseignement en Neurosciences; NINCDS-ADRDA, National Institute of Neurological and Communicative Disorders and Stroke and the Alzheimer's Disease and Related Disorders Association; DWRT, the Delayed Word Recall Test; DSST, the Digit Symbol Substitution Test; WFT, the Word Fluency Test; NR, Not reported; RCT, Randomized controlled trials; H&P, History and physical examination*.

### Study Quality

**Figure 2:**
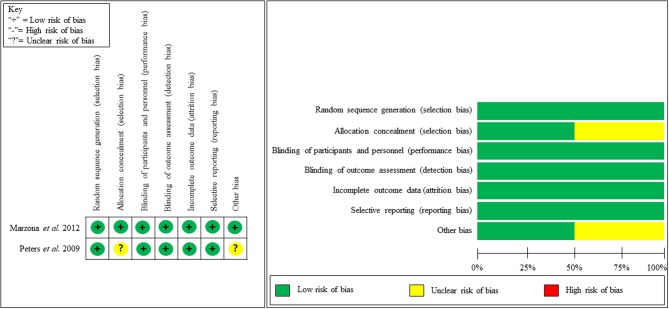
Methodological quality assessment of RCT.

Our quality assessment of each observational study (cohort study) was conducted using the NOS, which is specifically used to assess the methodological quality of non-randomized studies. We evaluated methodological quality in addressing patients' selection, study comparability, and the assessment of outcome or exposure. We then categorized studies into good, fair, or poor quality. Nine studies received good quality, four studies received fair quality and one study received poor quality (Table 2). Figure 2 shows the study quality for RCTs.

**Table 2:** Quality assessment using the Newcastle Ottawa Scale.

	**Selection (S)**				**Comparability (C)**		**Outcome**			**Subtotal assessment**			
	**1**	**2**	**3**	**4**	**1a**	**1b**	**1**	**2**	**3**	**S^+^**	**C^and^**	**E/O^and^**	**Conclusion**
**Cohort Studies**													
Tilvis et al. (2004)	^*^	^*^	^*^	^*^	^*^	^*^	No	No	^*^	Good	Fair	Fair	Fair
Elias et al. (2006)	^*^	^*^	^*^	^*^	^*^	No	^*^	No	^*^	Good	Fair	Good	Fair
Forti et al. (2006)	^*^	No	^*^	^*^	^*^	No	^*^	No	^*^	Good	Fair	Good	Fair
Rastas et al. (2007)	^*^	^*^	^*^	No	^*^	^*^	^*^	No	No	Good	Fair	Fair	Fair
Marengoni et al. (2011)	^*^	No	^*^	No	No	No	^*^	No	No	Good	Poor	Fair	Poor
Bunch et al. (2010)	^*^	^*^	No	^*^	^*^	^*^	^*^	No	^*^	Good	Good	Good	Good
Dublin et al. (2011)	^*^	^*^	No	^*^	^*^	^*^	^*^	No	^*^	Good	Good	Good	Good
Rusanen et al. (2014)	^*^	^*^	^*^	^*^	^*^	^*^	^*^	^*^	^*^	Good	Good	Good	Good
de Bruijn et al. (2015)	^*^	^*^	^*^	^*^	^*^	^*^	^*^	^*^	No	Good	Good	Good	Good
Liao et al. (2015)	^*^	^*^	^*^	^*^	^*^	^*^	^*^	^*^	^*^	Good	Good	Good	Good
Marzona et al. (2016)	^*^	^*^	^*^	^*^	^*^	^*^	^*^	^*^	^*^	Good	Good	Good	Good
Singh-Manoux et al. (2017)	^*^	^*^	^*^	^*^	^*^	^*^	^*^	^*^	^*^	Good	Good	Good	Good
Chen et al. (2018)	^*^	^*^	^*^	^*^	^*^	^*^	^*^	^*^	^*^	Good	Good	Good	Good
Ding et al. (2018)	^*^	^*^	^*^	^*^	^*^	^*^	^*^	^*^	^*^	Good	Good	Good	Good

+ *Domain scored: 0–1 (Poor); 2 (Fair); 3+ (Good)*.

and *Domain scored: 0 (Poor); 1 (Fair); 2+ (Good)*.

* *Domain acceptable*.

### Meta-Analysis

#### Primary Analysis

##### Atrial fibrillation and dementia risk

**Figure 3:**
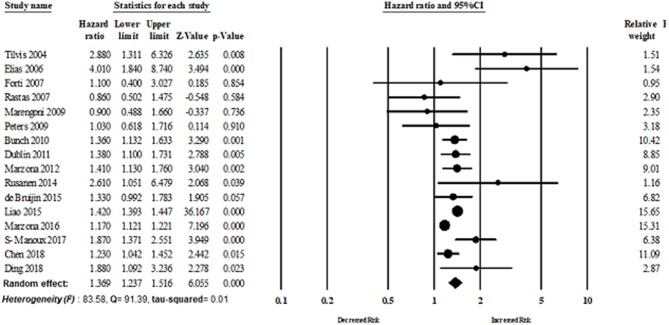
Association between AF and dementia risk.

Sixteen studies evaluated the magnitude of the association between AF and dementia risk. The overall adjusted pooled HR of developing dementia was 1.36 (95% CI: 1.23–1.51, *p* < 0.0001; *I*^2^ = 83.58), in patients with AF. Figure 3 shows the overall risk of dementia patients with AF.

#### Secondary Analysis

##### Atrial fibrillation and AD risk

**Figure 4:**
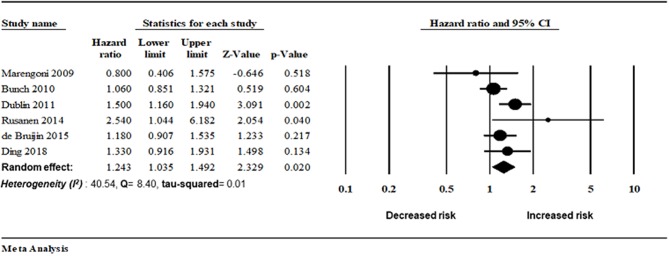
Association between AF and Alzheimer disease risk.

**Figure 5:**
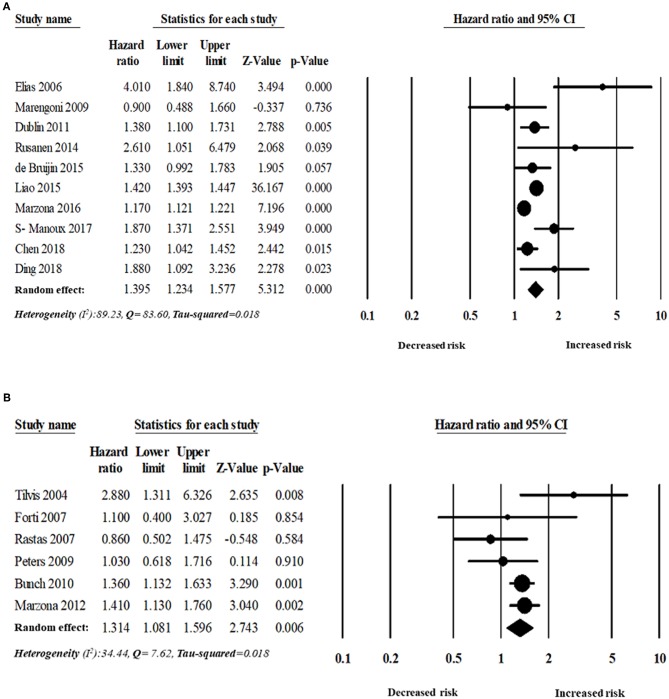
Risk of dementia in patients with AF based on follow-up period: **(A)** more than five years' follow-up period, **(B)** <5 years' follow-up period.

Six studies assessed the magnitude of the association between AF and AD risk. There was an increased risk of AD in patients with AF (random-effect HR: 1.24, 95% CI: 1.03–1.49, *p* = 0.02). However, lower heterogeneity was observed in this analysis (*I*^2^ = 40.54, Q = 8.40, tau^2^ = 0.019) (Figures 4, 5).

##### Subgroup analyses

The analyses were also stratified by the study design, region, and follow-up period. While stratified by the length of the follow-up period, the overall pooled HR was appeared to be higher in those studies with more than 5 years of follow-up (*n* = 10 studies). The overall adjusted pooled HR was 1.39 (95% CI:1.23–1.58, *p* < 0.0001) in the random effect model with higher heterogeneity (*I*^2^ = 89.23, Q = 83.60, tau^2^ = 0.018). However, the overall pooled risk of dementia was 31% for the study with <5 year of follow-up period (HR 1.31, 95% CI: 1.08–1.60, *p* = 0.006) in the random effect model with lower heterogeneity (*I*^2^ = 34.44, Q = 7.62, tau^2^ = 0.018).

**Figure 6:**
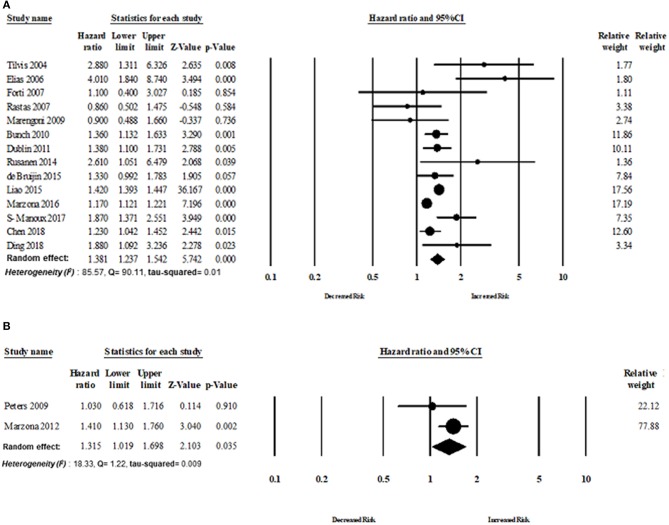
Association between AF and dementia based on study design: **(A)** cohort study design, **(B)** randomized control trial.

Fourteen cohort studies and two RCTs evaluated the risk of dementia in patients with AF. The overall risk of dementia of cohort studies was HR 1.38 (95% CI: 1.23–1.55, *p* < 0.0001) in the random effect model with higher heterogeneity (*I*^2^ = 85.57, Q = 90.11, tau^2^ = 0.018). The overall risk of dementia of RCT studies was HR 1.31 (95% CI: 1.01–1.70, *p* = 0.03) in the random effect model with lower heterogeneity (*I*^2^ = 18.33, Q = 1.22, tau^2^ = 0.009) (Figure 6).

**Figure 7:**
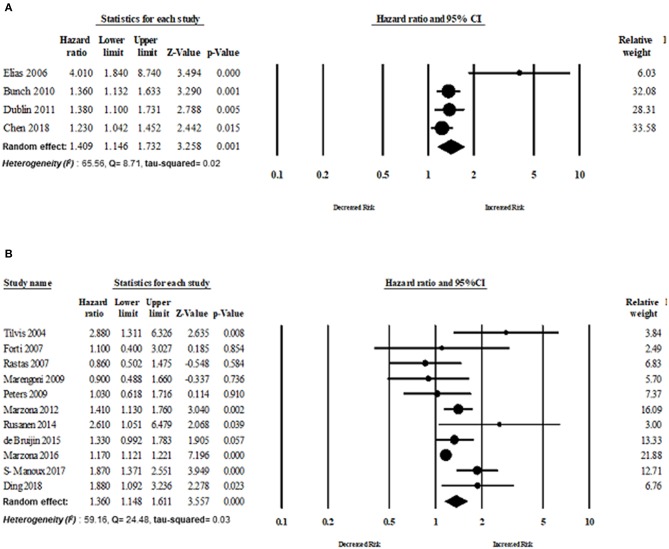
Risk of dementia based on region: **(A)** Europe, **(B)** North America.

Eleven studies published in Europe assessed the risk of dementia in patients with AF. The overall risk of dementia of studies published in Europe was HR 1.36 (95% CI: 1.14–1.61, *p* < 0.0001, *I*^2^ = 59.16, Q = 24.48, tau^2^ = 0.034). The overall risk of dementia of studies (*n* = 4) published in North America was HR 1.40 (95% CI: 1.14–1.73, *p* = 0.001, *I*^2^ = 65.56, Q = 8.71, tau^2^ = 0.026) (Figure 7).

However, the comparison was also stratified by good and fair methodological quality of study; the association of AF with dementia risk was significant in good methodological quality studies, but it appeared to be stronger in fair methodological quality studies (Supplementary Figure 1).

##### Publication bias

**Figure 8:**
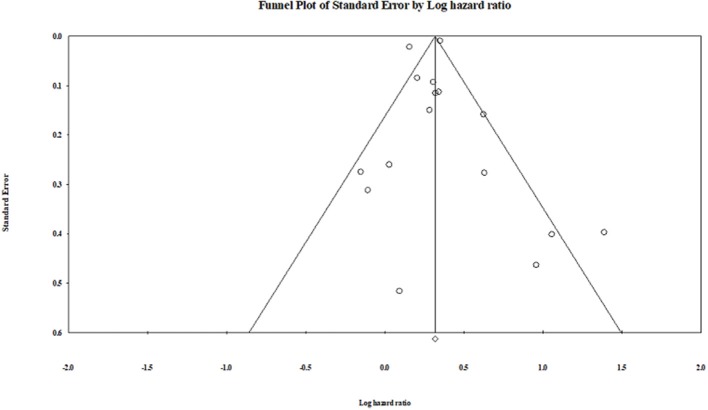
Funnel plot.

Publication bias was not observed in the pooled studies, or the subgroup analysis studies using the Egger test (Egger et al., 1997). The visual plot of the Egger regression test suggests that publication bias was unlikely (*P* = 0.90) (Figure 8).

## Discussion

### Main Findings

Our meta-analysis provides evidence for a significant association between AF and risk of dementia. This meta-analysis involves a total of 16 unique observational studies with aggregate data on 2,415,356 adult individuals, and approximately 200,653 cases of dementia followed up over a median period of 7.3 years. We found that the presence of AF is associated with an increased risk of dementia (1.36-fold). In addition, when the analysis was stratified either by follow-up duration, study country, or study design, the association between AF and dementia risk appeared to be stronger in studies with a follow-up duration longer than 5 years, and in studies conducted in Asia compared with those conducted in North America and Europe countries. The risk of dementia in AF patients was higher in the cohort studies compared with the one found for the RCT studies. Most of the included studies used MMSE to assess cognitive function, and dementia was clinically diagnosed according to the DSM-IV criteria. Moreover, published studies also diagnosed vascular dementia and AD according to the NINDS-AIREN (National Institute of Neurological Disorders and Stroke and Association Internationale pour la Recherch'e et l'Enseignement en Neurosciences) criteria and the NINCDS-ADRDA (National Institute of Neurological and Communicative Disorders and Stroke and the Alzheimer's Disease and Related Disorders Association) criteria, respectively.

### Comparison With Previous Studies

A previous meta-analysis (Santangeli et al., 2012) evaluated the prospective relationship between AF and incident dementia based on only eight studies. In addition, they did not perform a subgroup analysis and did not provide any biological evidence regarding AF and dementia risk. Therefore, their analyses provide a less robust basis for decision and policy making. Kwok et al. (2011) also conducted a meta-analysis with 15 relevant studies and demonstrated that AF was associated with a significant increase in dementia, but their study also lacked subgroup analyses. In our meta-analysis, we included a higher number of studies and provided a comprehensive analysis to support the current evidence.

### Biological Plausibility

**Figure 9:**
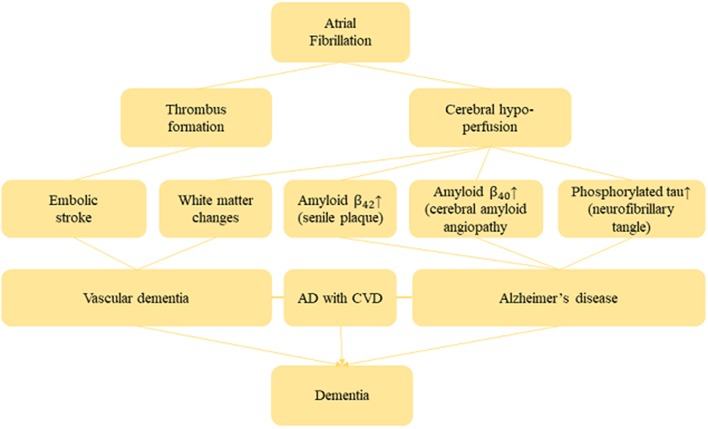
Biological mechanism between AF and dementia.

Several studies provided convincing evidence of the biological plausibility between AF and risk of dementia (Conway et al., 2004; Yaffe et al., 2014; Alosco et al., 2015). Indeed, AF may induce cerebral hypo-perfusion due to low cardiac output (Poggesi et al., 2015). However, cerebral hypo-perfusion exacerbates white matter change that is responsible for demyelination and axonal damage, and also leads to memory impairment (Shibata et al., 2004; Ihara and Tomimoto, 2011). In general, most vital organs in the human body including the brain have autoregulation mechanisms which maintain blood circulation even if cardiac output decreases (Akinyemi et al., 2013). However, this compensatory mechanism may deteriorate in a patient with long-term AF and thus lessen his cerebral circulation. Therefore, long-term cerebral hypo-perfusion induces senile plaques and amyloid angiopathy through β-(BACE 1) expression and γ-secretase. β- and γ-secretases then generates and aggregate β-amyloid peptide in the brain, which is always considered to be responsible for neuron death and AD (Pluta et al., 2013). Furthermore, senile plaques and cerebral amyloid angiopathy are formed due to Aβ_42_ with a higher aggregation ability which accumulates in the brain parenchyma, and Aβ_40_ with relatively lower aggregation ability accumulates in the cerebral blood vessels. Evidence from an *in-vivo* study shows that cerebral amyloid angiopathy (CA) triggers reduced blood supply in the brain, and also intensifies Aβ production due to dysfunction of the vascular smooth muscle (Niwa et al., 2002). It was also shown that CA hampers the Aβ clearance pathway, resulting in the accumulation of Aβ and generating a cycle of Aβ accumulation (Hawkes et al., 2011). Moreover, accumulation of Aβ causes neurofibrillary tangles which are mainly composed of phosphorylated tau, and thus finally promotes neuronal cell death (Hardy and Allsop, 1991). Yao et al. (2012) reported that chronic hypo-perfusion activates the tau phosphorylation enzymes glycogen synthase kinase-3 beta and cyclin-dependent kinase-5 which eventually induces the accumulation of Aβ. Figure 9 shows the plausible mechanism of AF and dementia risk.

### Public Health Implications

Our present meta-analysis of observational studies reported an evidence for the existence of a significant association between AF and risk of dementia. Inflammatory biomarkers and thromboembolism led to reduced and intermittent cerebral perfusion during arrhythmia or silent cerebral ischemia. It can cause chronic cerebral hypo-perfusion which is considered as a predictors of dementia (Jacobs et al., 2015). However, it should also be noted that oral anticoagulants (OACs) and a healthy lifestyle may reduce the risk of a dementia in patients with AF. Since patients with AF always tend to have a higher risk of strokes, they are consequently also at particular risk for cognitive decline, functional deficit and dependency. However, Ng et al. (2013) reported that novel anticoagulants, dabigatran, rivaroxaban, and apixaban are effective at reducing the absolute risk of stroke and are safer in elderly patients. Furthermore, an observational study provided interesting findings that included 2,685 dementia-free individuals from the Swedish National Study on Aging and Care in Kungsholmen, Stockholm. AF patients with OACs had a decreased risk of dementia (HR = 0.44, 95% CI: 0.18–0.92) compared to those patients without OACs (Ding et al., 2018). However, long-term anticoagulation therapy might raise the risk of intracranial bleeding. In addition, catheter ablation has emerged as a frontline therapy for improving symptoms, physical capacity, and quality of life in drug refractory AF patients. Bunch et al. (2011) enrolled 4,212 consecutive patients who underwent AF ablation, and 16,848 AF patients without ablation. AF ablation patients had a significantly lower risk of dementia (0.2% of the AF ablation patients) in comparison to AF patients without ablation (0.9% of the AF with no ablation) (Bunch et al., 2011). However, catheter ablation sometimes might itself lead to silent strokes and cognitive impairment (Shah et al., 2016). It is therefore important for the physicians to follow recommendations for performing ablation as well as management of AF patients before and after the procedure (Refaat et al., 2017). Furthermore, physicians should raise awareness of the risk of dementia in patients with AF, and recommend how to reduce the risk of dementia which could include the cessation of smoking, and the control of hypertension, obesity, diabetes, and sleep apnea. If any suspicion regarding cognitive decline or dementia is confirmed, physicians should conduct an objective assessment of cognitive function.

### Strengths and Limitations

Our meta-analysis has several limitations that should be mentioned. Although a random-effect model was used in our meta-analysis, the findings of this current and comprehensive meta-analysis should be explained with caution because of their high heterogeneity (*I*^2^ > 75%) in the overall main analysis. It is feasible to summarize that high heterogeneity is observed due to differences in sample size, methodological quality, demographic, and ethno-racial characteristics of the study populations, various covariate assessments, different length of follow-up periods, and types of dementia. All these possible sources of statistical heterogeneity were then systematically scrutinized and evaluated using stratified analyses. However, higher heterogeneity was observed between the studies when the primary analysis was conducted but it is notable to mention that there was very low heterogeneity as well as a greater significant association between AF and dementia risk when we stratified our analyses according to study design (RCTs) and different continents (North America and Europe).

Despite these limitations, this current and updated meta-analysis has several strengths. This current study provides the most comprehensive evaluation between AF and dementia risk so far. These findings were obtained from a larger number of participants and dementia cases from 16 observational studies (overall pooled risk of non-randomized and randomized are almost similar, showing higher risk of dementia with AF patients). Moreover, it is notable to point out that we applied standardized risk estimates from all relevant studies to embrace a harmonious combination of estimates across studies. The greater number of dementia patient's inclusion, stratification according to study design, region, methodological quality, and follow-up duration provided rational statistical power to quantitatively evaluate the association between AF and dementia risk. Finally, some sort of reported bias of the included studies were not a concern in our analyses, as our extensive literature search and discussion with main investigator made it unlikely that any published article was missed, and visual inspection of funnel plots showed no publication bias.

## Conclusion

The present comprehensive meta-analysis suggests that AF is associated with an increased risk of dementia. It is still unclear whether treatments of AF might lead the greater risk of dementia or not. Therefore, more biological studies are needed to find out their causality.

## Author Contributions

All authors listed have made a substantial, direct and intellectual contribution to the work, and approved it for publication.

### Conflict of Interest

The authors declare that the research was conducted in the absence of any commercial or financial relationships that could be construed as a potential conflict of interest.
