# Supplementary material for: Association Between Atrial Fibrillation and Dementia: A Meta-Analysis
Source: Front Aging Neurosci. 2019 Nov 8;11:305. doi: 10.3389/fnagi.2019.00305 (PMC6857071; doi:10.3389/fnagi.2019.00305)
Supplement: Supplementary file 1 [file Table_1.DOCX]

**Supplementary Table 1**: PRISMA 2009 Checklist

| **Section/topic** | **#** | **Checklist item** | **Reported on page #** |
| --- | --- | --- | --- |
| **TITLE** | | | |
| **Title** | **1** | Identify the report as a systematic review, meta-analysis, or both. | 1 |
| **ABSTRACT** | | | |
| Structured summary | **2** | Provide a structured summary including, as applicable: background; objectives; data sources; study eligibility criteria, participants, and interventions; study appraisal and synthesis methods; results; limitations; conclusions and implications of key findings; systematic review registration number. | 2 |
| **INTRODUCTION** | | | |
| Rationale | **3** | Describe the rationale for the review in the context of what is already known. | 3 |
| Objectives | **4** | Provide an explicit statement of questions being addressed with reference to participants, interventions, comparisons, outcomes, and study design (PICOS). | 3 |
| **METHODS** | | | |
| Protocol and registration | **5** | Indicate if a review protocol exists, if and where it can be accessed (e.g., Web address), and, if available, provide registration information including registration number. | N/A |
| Eligibility criteria | **6** | Specify study characteristics (e.g., PICOS, length of follow‐up) and report characteristics (e.g., years considered, language, publication status) used as criteria for eligibility, giving rationale. | 3 |
| Information sources | **7** | Describe all information sources (e.g., databases with dates of coverage, contact with study authors to identify additional studies) in the search and date last searched. | 3 |
| Search | **8** | Present full electronic search strategy for at least one database, including any limits used, such that it could be repeated. | 4 |
| Study selection | **9** | State the process for selecting studies (i.e., screening, eligibility, included in systematic review, and, if applicable, included in the meta‐analysis). | 4 |
| Data collection process | **10** | Describe method of data extraction from reports (e.g., piloted forms, independently, in duplicate) and any processes for obtaining and confirming data from investigators. | 4 |
| Data items | **11** | List and define all variables for which data were sought (e.g., PICOS, funding sources) and any assumptions and simplifications made | 4 |
| Risk of bias in individual studies | **12** | Describe methods used for assessing risk of bias of individual studies (including specification of whether this was done at the study or outcome level), and how this information is to be used in any data synthesis. | 4 |
| Summary measures | **13** | State the principal summary measures (e.g., risk ratio, difference in means). | 4 |
| Synthesis of results | **14** | Describe the methods of handling data and combining results of studies, if done, including measures of consistency (e.g., I2) for each meta‐analysis. | 4 |
| Risk of bias across studies | **15** | Specify any assessment of risk of bias that may affect the cumulative evidence (e.g., publication bias, selective reporting within studies). | 4 |
| Additional analyses | **16** | Describe methods of additional analyses (e.g., sensitivity or subgroup analyses, meta-regression), if done, indicating which were pre‐specified. | 4-5 |
| **RESULTS** | | | |
| Study selection | **17** | Give numbers of studies screened, assessed for eligibility, and included in the review, with reasons for exclusions at each stage, ideally with a flow diagram. | 5 |
| Study characteristics | **18** | For each study, present characteristics for which data were extracted (e.g., study size, PICOS, follow-up period) and provide the citations. | 5 |
| Risk of bias within studies | **19** | Present data on risk of bias of each study and, if available, any outcome level assessment (see item 12). | 5 |
| Results of individual studies | **20** | For all outcomes considered (benefits or harms), present, for each study: (a) simple summary data for each intervention group (b) effect estimates and confidence intervals, ideally with a forest plot. | 5 |
| Synthesis of results | **21** | Present results of each meta-analysis done, including confidence intervals and measures of consistency. | 5 |
| Risk of bias across studies | **22** | Present results of any assessment of risk of bias across studies (see Item 15). | 6 |
| Additional analysis | **23** | Give results of additional analyses, if done (e.g., sensitivity or subgroup analyses, meta-regression [see Item 16]). | 6 |
| **DISCUSSION** | | | |
| Summary of evidence | **24** | Summarize the main findings including the strength of evidence for each main outcome; consider their relevance to key groups (e.g., healthcare providers, users, and policy makers). | 6-7 |
| Limitations | **25** | Discuss limitations at study and outcome level (e.g., risk of bias), and at review-level (e.g., incomplete retrieval of identified research, reporting bias). | 8 |
| Conclusions | **26** | Provide a general interpretation of the results in the context of other evidence, and implications for future research. | 8 |
| **FUNDING** | | | |
| Funding | **27** | Describe sources of funding for the systematic review and other support (e.g., supply of data); role of funders for the systematic review. | N/A |

From: Moher D, Liberati A, Tetzlaff J, Altman DG, The PRISMA Group (2009). Preferred Reporting Items for Systematic Reviews and Meta-Analyses: The PRISMA Statement. PLoS Med 6(6): e1000097.doi:10.1371/journal.pmed1000097

For more information, visit: [www.prisma‐statement.org](http://www.prisma‐statement.org).

# **Supplementary Table 2:** Modified Newcastle-Ottawa Scale for Risk of Bias Assessment

| **Case Control Studies** | **Cohort Studies** |
| --- | --- |
| **Selection:**  1. Is the case definition adequate?   1. yes, with independent validation 2. yes, e.g., record linkage or based on self-reports 3. no description   2) Representativeness of the cases   1. consecutive or obviously representative series of cases 2. potential for selection biases or not stated   3) Selection of Controls   1. community controls 2. hospital controls 3. no description   4) Definition of Controls   1. no history of disease (endpoint) 2. no description of source | **Selection**  1) Representativeness of the exposed cohort   1. truly representative of the average adult in the community 2. somewhat representative of the average adult in the community 3. selected group of users e.g. ,nurses, volunteers 4. no description of the derivation of the cohort   2) Selection of the non-exposed cohort   1. drawn from the same community as the exposed cohort 2. drawn from a different source 3. no description of the derivation of the non-exposed cohort   3) Ascertainment of exposure   1. secure record (e.g., surgical records) 2. structured interview 3. written self-report 4. no description   4) Demonstration that outcome of interest was not present at study start   1. yes 2. no |
| **Comparability:**  1) Comparability of cases and controls on the basis of the design or analysis   1. study controls for age 2. study controls for any additional factor | **Comparability**  1) Comparability of cohorts on the basis of the design or analysis   1. study controls for age 2. study controls for any additional factor |
| **Exposure**  1) Ascertainment of exposure   1. secure record (e.g., pharmacy records) 2. structured interview where blind to case/control status 3. interview not blinded to case/control status 4. written self-report or medical record only 5. no description   2) Same method of ascertainment for cases and controls   1. yes 2. no   3) Non-Response rate   1. same rate for both groups 2. non respondents described 3. rate different and no designation | **Outcome**  1) Assessment of outcome   1. independent blind assessment 2. record linkage 3. self-report 4. no description   2) Adequacy of follow up of cohorts   1. complete follow up - all subjects accounted for 2. subjects lost to follow up unlikely to introduce bias: i.e.,<10% small number, lost, or description provided of those lost 3. follow-up rate < 90% and no description of those lost 4. no statement |

**Supplementary table 3:**

Baseline patient with different covariates:

| Covariates | Tilvis | Elias | Forti | Rastas | Marengoni | Peters | Bunch | Dublin | Marzona | Rusanen | Liao | de Bruijn | Marzona | S- Manoux | Ding | Chen |
| --- | --- | --- | --- | --- | --- | --- | --- | --- | --- | --- | --- | --- | --- | --- | --- | --- |
| Mean age of AF patients | 75 | 68.1 | 79.3 | 89.0 | 78 | 60 | 68.1 | 76.5 | 66.5 | 51.3 | 70.3 | 75.7 | 78.40 | 58.3 | 80.9 | 59.3 |
| Age | √ | √ | √ | √ | √ |  | √ | √ | √ | √ | √ | √ | √ |  | √ | √ |
| Gender | √ |  | √ | √ | √ |  | √ | √ |  | √ | √ | √ | √ | √ | √ | √ |
| History of stroke | √ |  |  | √ |  | √ |  |  | √ | √ |  |  | √ |  | √ | √ |
| History of myocardial infarction | √ |  |  | √ |  |  | √ |  | √ |  |  |  | √ |  |  |  |
| Atrial fibrillation | √ |  | √ |  |  | √ | √ |  |  |  |  |  |  |  |  |  |
| Intermittent claudication | √ |  |  |  |  |  |  |  |  |  |  |  |  |  |  |  |
| Hypertension | √ |  |  | √ | √ |  | √ | √ | √ |  | √ |  |  | √ | √ |  |
| Cerebral vascular accident |  |  |  |  |  |  | √ |  |  |  | √ |  | √ |  |  |  |
| Diabetes mellitus | √ | √ |  | √ |  | √ | √ | √ | √ | √ | √ | √ | √ | √ | √ | √ |
| Coronary heart disease |  |  |  |  |  |  |  | √ |  |  | √ | √ |  |  | √ | √ |
| Plasma insulin | √ |  |  |  |  |  |  |  |  |  |  |  |  |  |  |  |
| COPD |  |  |  |  |  |  |  |  |  |  | √ |  |  |  |  |  |
| Autoimmune disease |  |  |  |  |  |  |  |  |  |  | √ |  |  |  |  |  |
| Malignancy |  |  |  |  |  |  |  |  |  |  | √ |  |  |  |  |  |
| Dyslipidemia |  |  |  |  |  |  |  |  |  |  | √ |  |  |  |  |  |
| C-reactive protein | √ |  |  |  |  |  |  |  |  |  |  |  |  |  |  |  |
| Serum cholesterol | √ |  |  |  |  |  |  |  |  |  |  |  |  |  |  |  |
| APOE4 | √ |  |  | √ | √ |  |  | √ |  | √ |  | √ |  |  | √ | √ |
| Serum Ca^++^ | √ |  |  |  |  |  |  |  |  |  |  |  |  |  |  |  |
| Feelings of loneliness | √ |  |  |  | √ |  |  |  |  |  |  |  |  |  |  |  |
| Education |  | √ | √ | √ |  | √ |  | √ | √ | √ |  | √ |  | √ | √ | √ |
| Systolic blood pressure, mm Hg |  | √ |  |  |  | √ |  | √ | √ | √ |  |  |  |  |  | √ |
| Diastolic blood pressure, mm Hg |  | √ | √ |  |  |  |  | √ | √ |  |  |  |  |  |  | √ |
| Alcohol, ml/week |  | √ |  |  |  |  |  |  | √ |  |  |  |  | √ | √ |  |
| Cigarettes/ day |  | √ |  |  |  |  |  |  |  |  |  |  |  |  |  |  |
| Total cholesterol |  | √ |  |  |  | √ |  |  |  | √ |  | √ |  |  | √ |  |
| BMI (kg/m^2^) |  | √ | √ |  |  |  |  | √ | √ | √ |  | √ |  |  | √ | √ |
| Depressed mood (CESD>16) |  | √ |  |  |  |  |  |  |  |  |  |  |  |  |  |  |
| History of cardiovascular disease |  | √ |  |  |  |  |  | √ |  |  |  | √ | √ |  |  |  |
| ECG left ventricular hypertrophy |  | √ |  |  |  |  |  |  |  |  |  |  |  |  |  |  |
| Coronary artery bypass graft surgery |  | √ |  |  |  |  |  |  |  |  |  |  |  |  |  |  |
| Treatment with antihypertensive drugs |  | √ |  |  |  | √ |  |  |  |  |  |  |  |  |  | √ |
| Treatment of anticoagulant drugs |  | √ |  |  |  |  |  |  | √ |  |  | √ | √ |  |  | √ |
| Treatment of antiplatelet therapy |  |  |  |  |  |  |  |  | √ |  |  |  | √ |  |  | √ |
| Treatment of antiarrhythmic drugs |  |  |  |  |  |  |  |  |  |  |  |  | √ |  |  |  |
| Treatment with digitalis |  | √ |  |  |  |  |  |  |  |  |  |  |  |  |  |  |
| Treatment with beta-blockers |  | √ |  |  |  |  | √ |  |  |  |  |  |  |  |  |  |
| Antithrombotic medication |  |  |  |  | √ |  |  |  | √ |  |  |  |  |  |  |  |
| Angiotensin receptor blocker |  |  |  |  |  |  | √ |  |  |  |  |  |  |  |  |  |
| Diuretic |  |  |  |  |  |  | √ |  |  |  |  |  |  |  |  |  |
| Serum folate (nmol/l) |  |  | √ |  |  |  |  |  |  |  |  |  |  |  |  |  |
| ACE inhibitor |  |  |  |  |  |  | √ |  |  |  | √ |  |  |  |  |  |
| Statin |  |  |  |  |  |  | √ |  | √ |  | √ | √ | √ | √ |  |  |
| Ramipril |  |  |  |  |  |  |  |  | √ |  |  |  |  |  |  |  |
| Telmisartan |  |  |  |  |  |  |  |  | √ |  |  |  |  |  |  |  |
| MMSE |  |  | √ | √ | √ |  |  |  | √ |  |  |  |  |  | √ |  |
| MCI subtype |  |  | √ |  |  |  |  |  |  |  |  |  |  |  |  |  |
| aMCI |  |  | √ |  |  |  |  |  |  |  |  |  |  |  |  |  |
| Heart failure |  |  |  | √ |  | √ | √ | √ |  |  |  | √ | √ | √ | √ | √ |
| Warfarin |  |  |  | √ |  |  |  | √ |  |  | √ |  |  |  |  |  |
| Creatinine (mmol/l) |  |  |  |  |  | √ |  |  | √ |  |  |  |  |  |  |  |
| Glucose (mmol/l) |  |  |  |  |  | √ |  |  |  |  |  |  |  |  |  |  |
| Hemoglobin (g/dl) |  |  |  |  |  | √ |  | √ |  |  |  |  |  |  |  |  |
| Smoking |  |  |  |  |  |  | √ | √ | √ | √ |  | √ |  | √ | √ | √ |
| Ejection fraction |  |  |  |  |  |  | √ |  |  |  |  |  |  |  |  |  |
| Exercise/ level of physical activity |  |  |  |  |  |  |  | √ | √ | √ |  |  |  | √ | √ |  |
| Clopidogrel |  |  |  |  |  |  |  |  |  |  | √ |  |  |  |  |  |
| Aspirin |  |  |  |  |  |  |  |  |  |  | √ |  |  |  |  |  |
| Income level |  |  |  |  |  |  |  |  |  |  | √ |  |  |  |  |  |

**Supplementary Table 4:** Search strategy

| **Key words** | **MeSH term** |
| --- | --- |
| Atrial Fibrillation | ("atrial fibrillation"[MeSH Terms] OR ("atrial"[All Fields] AND "fibrillation"[All Fields]) OR "atrial fibrillation"[All Fields])atrial[All Fields] AND fibrillation[All Fields] |
| Dementia  Alzheimer disease  Vascular dementia | dementia[All Fields] AND risk[All Fields] AND case[All Fields] AND control[All Fields] AND study[All Fields] AND dementia[Title] AND risk [Title] AND cohort[Title] AND study[Title] AND dementia[All Fields] AND risk[All Fields] AND randomized[All Fields] AND control[All Fields] AND study[All Fields] AND ("alzheimer disease"[MeSH Terms] OR ("alzheimer"[All Fields] AND "disease"[All Fields]) OR "alzheimer disease"[All Fields]) AND ("risk"[MeSH Terms] OR "risk"[All Fields]) AND ("case-control studies"[MeSH Terms] OR ("case-control"[All Fields] AND "studies"[All Fields]) OR "case-control studies"[All Fields] OR ("case"[All Fields] AND "control"[All Fields] AND "study"[All Fields]) OR "case control study"[All Fields]) AND ("alzheimer disease"[MeSH Terms] OR ("alzheimer"[All Fields] AND "disease"[All Fields]) OR "alzheimer disease"[All Fields]) AND ("risk"[MeSH Terms] OR "risk"[All Fields]) AND ("cohort studies"[MeSH Terms] OR ("cohort"[All Fields] AND "studies"[All Fields]) OR "cohort studies"[All Fields] OR ("cohort"[All Fields] AND "study"[All Fields]) OR "cohort study"[All Fields]) |
