# Supplementary material for: Association Between Atrial Fibrillation and Dementia: A Meta-Analysis
Source: Front Aging Neurosci. 2019 Nov 8;11:305. doi: 10.3389/fnagi.2019.00305 (PMC6857071; doi:10.3389/fnagi.2019.00305)
Supplement: Supplementary file 2 [file Table_2.DOCX]

Supplementary Figure 1:

A.

B.

Figure 1: Association between AF and dementia based on a) good and b) fair methodological quality study
